# Effectiveness of a co-adapted virtual discharge education app on disease knowledge and health behaviours in patients following heart attack: a multicentre, randomised controlled trial protocol in Sydney, Australia

**DOI:** 10.1136/bmjopen-2025-114569

**Published:** 2026-02-18

**Authors:** Ling Zhang, Wendan Shi, Emma Zhao, Karice K Hyun, Robert Zecchin, Yan Gao, Ludimila Brunorio, Fiona Stanaway, Tiffany Ellis, Julie Redfern, Robyn Clark, Huiyun Du, Robyn Gallagher

**Affiliations:** 1 Faculty of Medicine and Health, The University of Sydney, Camperdown, New South Wales, Australia; 2 Sydney Nursing School, The University of Sydney, Sydney, New South Wales, Australia; 3 Faculty of Medicine and Health, The University of Sydney, Sydney, New South Wales, Australia; 4 Cardiology Department, ANZAC Research Institute, Concord Repatriation General Hospital, Concord, New South Wales, Australia; 5 Cardiac Rehabilitation Services, Westmead Hospital, Westmead, New South Wales, Australia; 6 Cardiology Department, St George Hospital, Kogarah, New South Wales, Australia; 7 Cardiology Department, Royal Prince Alfred Hospital, Camperdown, New South Wales, Australia; 8 School of Public Health, University of Sydney, Sydney, New South Wales, Australia; 9 Physiotherapy, Westmead Hospital, Westmead, New South Wales, Australia; 10 Bond University Institute for Evidence-Based Healthcare, Robina, Queensland, Australia; 11 School Nursing and Midwifery, Flinders University, Adelaide, South Australia, Australia; 12 Flinders University, Adelaide, South Australia, Australia

**Keywords:** acute coronary syndrome, coronary heart disease, patient discharge, randomised control trial, patient education as topic, emigrants and immigrants

## Abstract

**Introduction:**

Active self-management by patients following acute coronary syndrome (ACS) can reduce recurrent events. Patient education for transitioning from hospital to home promotes effective self-management but can be limited in the acute setting due to time and resource pressures. Patients from ethnic minority and immigrant backgrounds face additional language, cultural and health literacy barriers to receiving patient education. Self-administered virtual patient education presents an innovative solution to these challenges. This study aims to evaluate a co-adapted, virtual avatar nurse-guided, discharge education application (app) for Chinese-speaking patients following ACS.

**Methods and analysis:**

This multicentre, assessor-blinded, randomised controlled trial will recruit 98 Chinese-speaking inpatients following ACS with evaluation at 1 and 3 months postdischarge. Control participants in the control group will receive the usual ward-based patient discharge education. Intervention participants will additionally receive the education app installed on their devices before hospital discharge with unlimited access during the study period. Cultural relevance and linguistic accuracy for this Chinese version of an existing app were ensured through co-adaptation with Chinese-speaking consumers; the primary outcome will be coronary heart disease (CHD) knowledge, and secondary outcomes will include knowledge, attitudes and beliefs regarding heart attack symptoms and responses, CHD self-management behaviours, utilisation of healthcare services and quality of life. A process evaluation will be conducted alongside the trial to assess the acceptability and feasibility of the app. Between-group comparisons will be made using 95% CIs, accounting for baseline differences using linear mixed effects or mixed effects logistic regression models.

**Ethics and dissemination:**

The Western Sydney Local Health District Human Research Ethics Committee has approved this study protocol (26 February 2024, amendment number 2) (2024/STE00147), with site-specific authorisations obtained from each participating hospital. The results will be disseminated through peer-reviewed journal articles and presentations at scientific conferences.

**Trial registration number:**

ACTRN12624000408583.

**STRENGTHS AND LIMITATIONS OF THIS STUDY**

- This is the first multicentre design randomised controlled trial to evaluate a co-adapted virtual discharge education app for patients following a heart attack in Australia.
- The intervention is linguistically and culturally co-adapted with consumers, delivered digitally, allowing scalability and accessibility for diverse patient populations.
- Mixed quantitative and qualitative process evaluation provides insights for future implementation of the intervention.
- Blinding of participants and clinicians is not possible due to the nature of the intervention, which may introduce performance bias.
- The study is limited to hospitals in metropolitan Sydney, which may affect external validity for other regions or healthcare systems.

## Introduction

 Coronary heart disease (CHD) is a leading cause of death and disease burden globally. The incidence and prevalence of CHD are projected to increase by 116% and 106%, respectively, by 2050.^1^ Despite advancements in medical technology that have improved the survival rates from coronary events, 20%–30% of patients are rehospitalised within 30 days of discharge,^2^ and >30% die within 18 months of the initial event.^3^ These statistics emphasise the critical importance of secondary prevention in reducing readmissions and overall health burdens. Additionally, heart attack survivors often experience heightened fear of recurrence and uncertainty, contributing to unnecessary distress and slower recovery.^4^

Secondary prevention of CHD is often suboptimal,^5^ particularly among patient groups from ethnic minority and immigrant backgrounds. Global migration is increasing and magnifying this issue. Chinese immigrants represent the third-largest immigrant population globally^6^ and for those living in Western countries, there are poorer CHD secondary prevention and outcomes. The Chinese-born population is the fastest-growing overseas-born group in Australia, reaching 655 790 people, a 51.7% increase in the last 10 years.^7^ This growth places it as the third largest migrant group, after the UK and India, and the largest migrant group from non-English-speaking background. A meta-analysis has found mortality rates are 30% and 80% higher than those of white and South Asian populations.^8^ Mortality and recurrence are potentially preventable if patients understand the condition, possess self-management skills and actively engage in secondary prevention behaviours.^9^

Patient education, starting predischarge, is fundamental to success in secondary prevention. Extensive evidence has demonstrated that patient education can reduce cardiovascular risk factors, mortality and morbidity.^10^,^12^ Despite this, many immigrant patients, including Chinese immigrants, do not benefit fully from these strategies due to inequitable accessibility. Language barriers are particularly significant for older Chinese immigrants. For instance, in Australia, approximately 70% of older Chinese immigrants report inadequate English proficiency.^13^ This percentage with limited proficiency increases with age and is a barrier to using mainstream patient education programmes, predominantly delivered in English.^14 15^

Cultural differences and low health literacy pose additional challenges to accessing education programmes and learning.^13 16^ Most patient education programmes fail to consider these specific needs of immigrant patients,^17^ leading to significant barriers in accessing healthcare services.^18^ Although there are translated materials, quality is often inconsistent, and there is a lack of cultural relevance to Chinese-speaking patients.^17^ The absence of a systematic and rigorous approach to developing culturally and linguistically appropriate education resources has been noted as a significant gap in the current health literature.^19 20^ The effectiveness of education interventions is also limited by a lack of research and consumer engagement in developing materials addressing the unique needs of Chinese immigrants.

To address these issues and better engage Chinese-speaking populations, culturally and linguistically appropriate patient education interventions that accommodate lower health literacy levels of patients are urgently needed.^21^ One promising approach is the use of avatar technology, which combines computer-generated imagery and practical effects to communicate in natural ways.^22^ Avatars, which are simulated human characters, can engage users through facial expressions, body language, speech and natural language understanding.^23 24^ The technology has been successfully implemented in education interventions for patients managing heart failure,^24^ diabetes mellitus,^25^ mental health^26^ and obesity.^27^

Despite its potential, few interventions using avatar technology have been adapted and evaluated rigorously in Chinese-speaking patients with CHD. This study aims to address this gap by testing a linguistically and culturally co-adapted virtual nurse-guided discharge education app specifically designed for Chinese-speaking patients with ACS.

## Methods and analysis

### Study design

**Figure 1:**
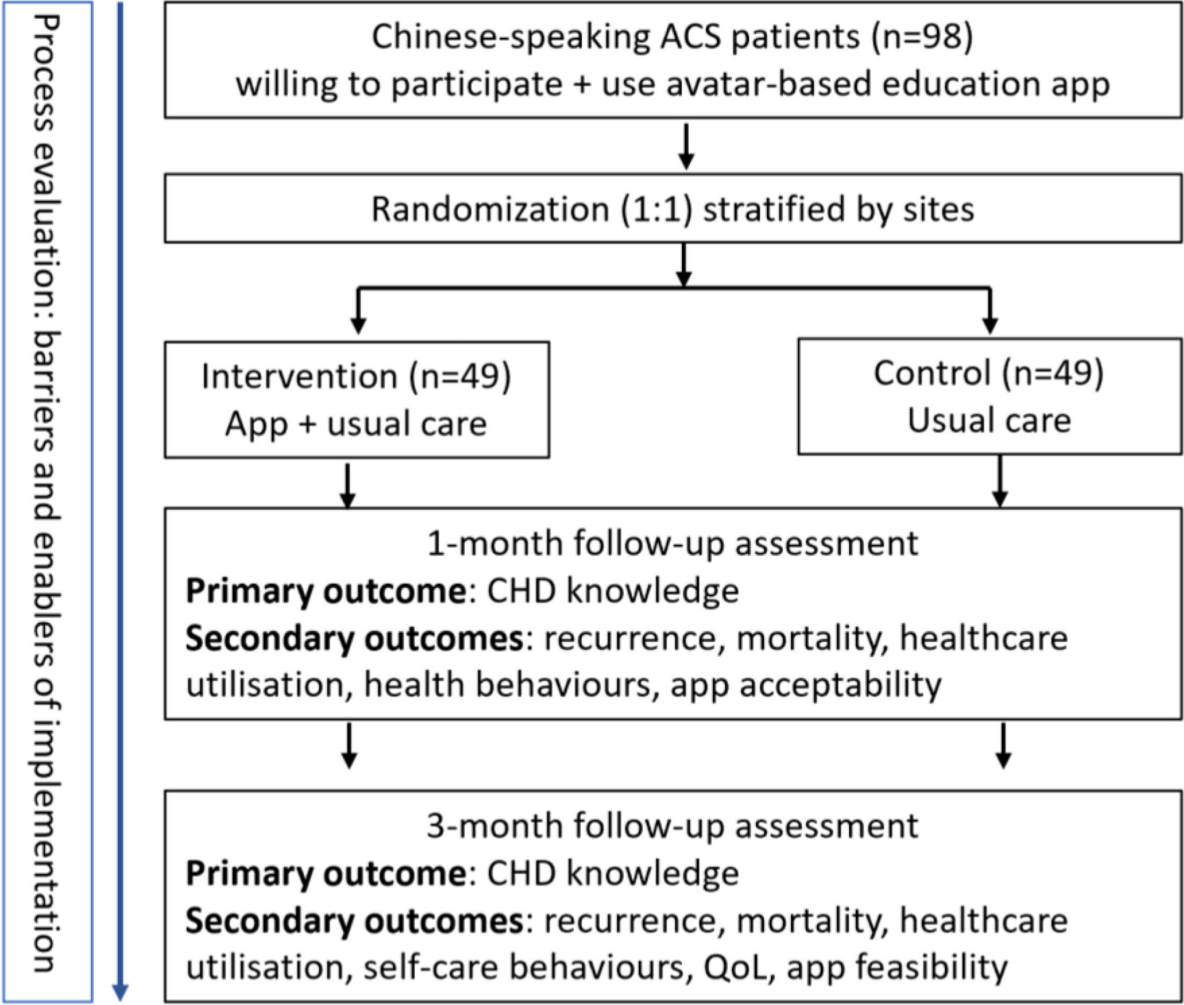
Overall project flow diagram. CHD, coronary heart disease; QoL, quality of life.

This study is a prospective, outcome assessor-blinded, multicentre, randomised controlled trial (RCT) of a discharge education app for Chinese-speaking patients with ACS that uses a virtual avatar nurse to guide users (figure 1). The trial will compare this app plus usual care with usual care alone in Chinese-speaking patients with ACS. A process evaluation will be conducted following the RCT. This study protocol was developed following the Standard Protocol Items: Recommendations for Interventional Trials Checklist.^28^ The trial has been prospectively registered with the Australia and New Zealand Clinical Trial Registry (ACTRN12624000408583).

### Setting and recruitment

This study will be conducted at three large tertiary public hospitals in metropolitan Sydney, Australia, which serve large populations with diverse social, economic and cultural backgrounds. China-born residents rank among the top overseas-born groups in the catchment area of these hospitals. The care provided in these hospitals is funded by Medicare-Australia’s universal health insurance scheme.

Cardiology ward staff who are not part of the research team will screen the admission lists of the wards at the participating hospitals to identify potential participants. A bilingual researcher will approach the potential participants to explore their interest in participating. Following informed consent, patients will be enrolled in the study and complete a baseline assessment by the bilingual researcher before randomisation. The baseline assessment will include participants’ demographic (age, gender, education, English proficiency, health literacy, marriage status, employment status, technology use) and clinical data (admission diagnosis, comorbidities). Patients who are ineligible or decline to participate will be recorded, and the reason for exclusion or refusal will be documented. Recruitment is anticipated to take place between 1 July 2024 and 31 August 2026.

### Participants

Inclusion criteria are:

1. adults (≥18 years) admitted to the participating hospitals with a diagnosis of ACS (unstable angina, non-ST-elevation and ST-elevation myocardial infarction);
2. being discharged home;
3. Chinese (Mandarin)-speaking;
4. having a personal smartphone/tablet computer with software capable of downloading the app.

Exclusion criteria are:

1. critically unwell or have severe co-existing medical conditions that would impede or prevent engagement in lifestyle behaviour change, for example, dementia or terminal illness;
2. unable to understand, speak or read basic Chinese to consent;
3. sufficient vision, manual dexterity or hearing to use the app.

### Randomisation and blinding

Participants will be randomly allocated to either the intervention or control group after the baseline assessment is completed. Randomisation will be performed by a research team member independent of recruitment, data collection and analysis using Excel functions with a 1:1 ratio (intervention:control), who will also be responsible for securely keeping the allocation.

Follow-up assessors will be blinded to group allocation with no anticipated circumstances for which unblinding is required. Health professionals delivering usual care will remain blinded to the participant’s group allocation unless the participant discloses it. Due to the nature of the intervention, blinding the intervention to participants is not possible.

### Study groups

#### Control

As determined by the hospitals, participants in the control group will receive usual discharge education from their doctors and other healthcare professionals, such as nurses or pharmacists. This discharge education is usually delivered by a Chinese-speaking health professional or through an English-speaking family member and provides information that summarises discharge, medications and follow-up care, such as cardiac rehabilitation. At the conclusion of the study, control participants will receive a link to download the app.

#### Intervention

After baseline assessment and randomisation, a researcher who completes the baseline assessment will assist participants allocated to the intervention group with downloading and installing the app on their smartphone or tablet before hospital discharge. The researcher will ensure that the participants are familiar with the app and its functions and have access to it throughout the study period, in addition to their usual care. The participants will have unlimited independent access to all the educational modules. They will be encouraged to contact the researcher if they experience technical issues with the app during the study period.

### Intervention details

#### Intervention content

The education app, called ‘6 Steps to Cardiac Recovery’, for heart attack survivors was codesigned and developed in English.^29^ The app content includes six modules of evidence-based information recommended by international and Australian national guidelines for heart attack survivors and their families.^30^,^32^ The modules cover diagnosis/procedures, heart disease risk factors, cardiac rehabilitation, medications, heart attack warning signs and actions and ongoing medical review. Completing all six modules takes approximately 40 min. A virtual nurse, created using avatar technology, guides the user through the six modules and promotes engagement. Interactive questions, quizzes and games are used throughout the app to promote interest, and the virtual nurse provides instant responses based on the correctness of users’ responses.

#### Intervention co-adaptation process

The original app is likely less beneficial for patients from immigrant backgrounds who are not proficient in English and may have culturally specific needs. Therefore, this app was adapted to Chinese language and culture using a co-adaptation process with Chinese-speaking consumers. The process followed principles commonly used in cultural adaptation for psychosocial interventions to ensure that the content, context and delivery were tailored to the Chinese-speaking population.

**Figure 2:**
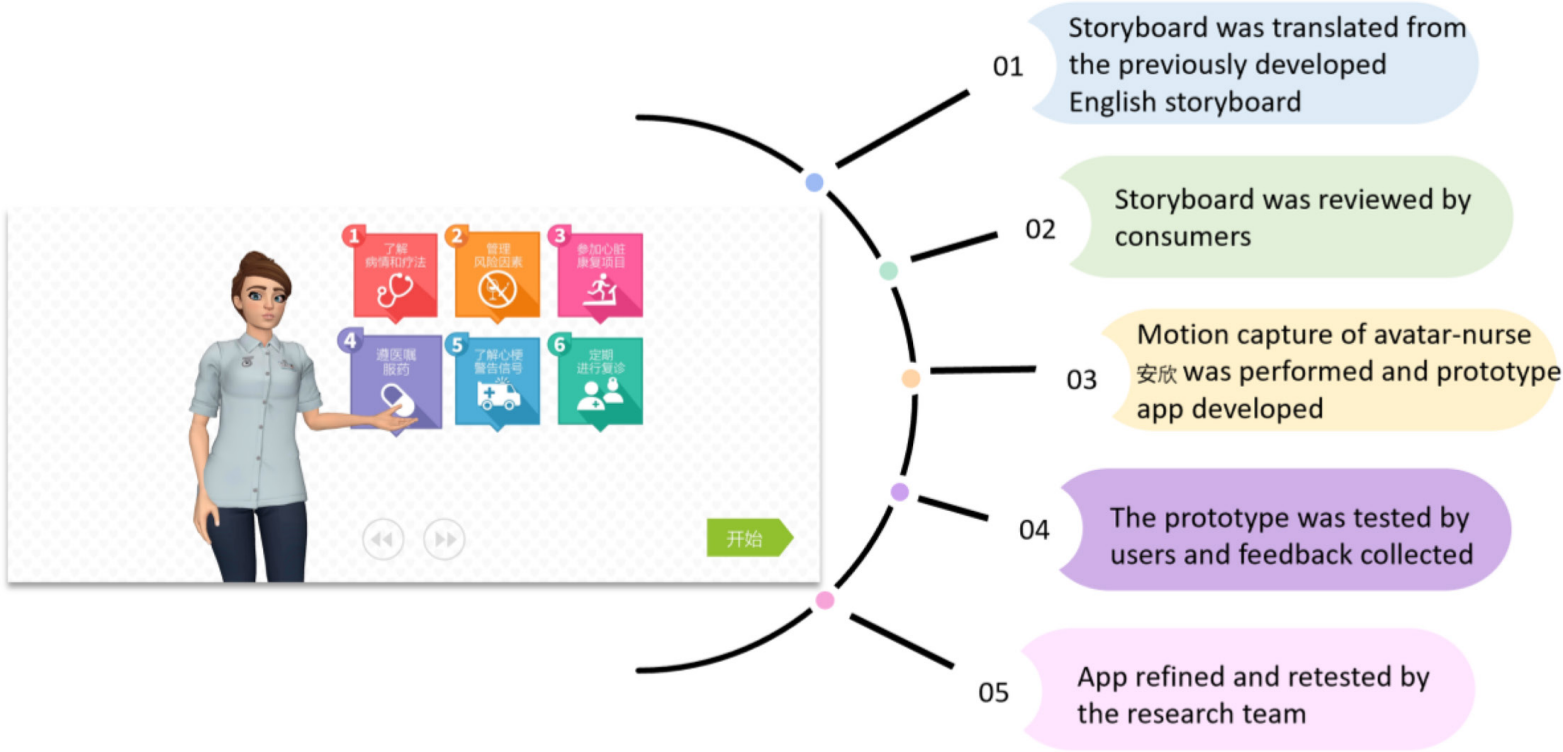
Co-adaptation process of the virtual nurse-guided education app for Chinese-speaking patients.

Five steps were involved in co-adaptation, including (1) creating storyboards in Chinese; (2) storyboard review by consumers to ensure language and culture appropriateness; (3) developing an avatar-based prototype; (4) pretesting the prototype by Chinese-speaking consumers and (5) refining and retesting the app. Chinese-speaking patients (n=3) and bilingual clinicians (n=3) were substantially engaged in steps II and IV to ensure language accuracy, readability, understandability, clarity and cultural sensitivity (figure 2).

#### Intervention features

This simplified Chinese version of the app fully addresses Chinese-speaking patients’ language, culture, health literacy and technology literacy needs. Simple language and multiple visual methods are used to improve readability and understandability, and questions and games were incorporated to enhance engagement based on evidence.^33^ The virtual nurse’s Chinese name is Anxin (安欣), whose facial expressions were created using a Chinese model. Anxin interacts with users through natural and innate human communication modalities (facial expressions, body language, speech and natural language understanding), which are illustrated in the screenshots of the app (online supplemental images 1 and 2).

### Data collection and study outcomes

Assessments will occur at baseline and 1 and 3 months after hospital discharge through phone interviews conducted by a researcher, as listed in table 1.

**Table 1:** Study outcomes and assessment time point

Study outcome	Measurement	Baseline	1 month	3 months
**Primary outcome**				
CHD knowledge	CADE-Q SV (Simplified Chinese)	✓	✓	✓
Knowledge, attitudes and beliefs about heart event	C-ACSRI	✓	✓	✓
**Key secondary outcomes**				
Self-management behaviours	CADSMS	✓		✓
Cardiac-related hospital admission	Self-reported or family reported		✓	✓
Healthcare service utilisation	Self-reported		✓	✓
**Other secondary outcomes**				
Cardiac-related mortality	Family reported		✓	✓
Quality of life	EQ-5D-5L	✓		✓
App acceptability	App user survey		✓	
App feasibility	Data analytics			✓
Barriers and facilitators of implementation	Individual interview and focus group			✓

C-ACSRI, Chinese version of Acute Coronary Syndrome Response Index; CADE-Q SV, short version of the Chinese-language Coronary Artery Disease Education Questionnaire; CADSMS, Coronary Artery Disease Self-Management behaviour Scale; CHD, coronary heart disease; EQ-5D-5L, Five-Level EuroQol five-dimension instrument.

The primary outcome will be the mean difference between intervention and control groups in CHD knowledge scores and knowledge, attitudes and beliefs about heart attack symptoms and responses. Key secondary outcomes will include CHD self-management behaviours, cardiac-related hospital admission and use of healthcare services. Other secondary outcomes will include cardiac-related mortality, quality of life, app acceptability, app feasibility and barriers and facilitators of implementation.

#### Coronary heart disease knowledge

CHD knowledge will be measured using the short version of the Chinese-language Coronary Artery Disease Education Questionnaire (CADE-Q SV) at 1 and 3 months after hospital discharge.^34^ This 20-item questionnaire assesses participants’ overall CHD knowledge and five subdomains related to disease, risk factors, exercise, nutrition and psychological aspects, with four items in each subdomain. Each item is scored based on a ‘true’, ‘false’ or “I don’t know” response. Each correct answer receives 1 point for a maximum score of 20 for overall knowledge and 4 points for each domain. The CADE-Q SV has been validated among Chinese immigrants with CHD.^34^

#### Knowledge, attitudes and beliefs about heart attack symptoms and responses

Participants’ knowledge, attitudes and beliefs about heart attack symptoms and responses will be measured using the Chinese version of the ACS Response Index at 1 and 3 months posthospital discharge. The measure comprises three subdomains: heart attack knowledge, attitudes and beliefs. The 21-item knowledge subdomain is scored based on dichotomous responses (yes/no) to a statement, with each correct response receiving 1 point, for a maximum score of 21. The 5-item attitudes subdomain is scored on a 4-point Likert scale, ranging from ‘not at all’ (1 point) to ‘very sure’ (4 points), for a maximum score of 20. The 7-item beliefs subdomain is scored on a 4-point Likert scale, ranging from ‘strongly agree’ (1 point) to ‘strongly disagree’ (4 points), for a maximum score of 28. The questionnaire has been translated and validated in Chinese patients with cardiac conditions.^35^

#### Self-management behaviours

Participants’ CHD self-management behaviours will be measured 3 months posthospital discharge using the Coronary Artery Disease Self-Management behaviour Scale. This 27-item questionnaire has three subdomains, including emotional self-management behaviours, daily life self-management behaviours and medical self-management behaviours. Participants rate each item on a 5-point Likert scale, for a total score ranging from 27 to 135, with a higher score indicating better self-management behaviours. The questionnaire was originally developed and validated in Chinese patients with CHD.^36^

#### Cardiac-related hospital admission

Cardiac-related hospital admission will be collected from participants’ self-reported data and cross-referenced with electronic medical records.

#### Healthcare service utilisation

Healthcare use, including cardiac rehabilitation attendance and other healthcare services (regular planned and/or unplanned visits to their general practitioners, a chronic disease management plan, enrolment in a health coaching service and weight loss programmes), will be collected from participants’ self-reported data and cross-referenced with electronic medical records.

#### Cardiac-related mortality

Cardiac-related mortality will be based on family reported data and cross-referenced with hospital electronic medical records.

#### Quality of life

Quality of life will be measured 3 months posthospital discharge using the Five-Level EuroQol five-dimension instrument (EQ-5D-5L) (Simplified Chinese).^37^ Participants will be assessed across five health dimensions, including mobility, self-care, usual activities, pain/discomfort and anxiety/depression, based on five response categories from level 1 to 5 corresponding with no problem, slight problems, moderate problems, severe problems and extreme problems, respectively.^38^ Alongside the five health dimensions, the EQ visual analogue scale will be used to assess participants’ ‘own health today’ with end points labelled ‘best imaginable health state’ (100) and ‘worst imaginable health state’ (0). The EQ-5D-5L has been validated in Chinese populations.^37^

#### Process outcomes and measures

Alongside the clinical trial, a process evaluation will be conducted to explore the acceptability, feasibility and barriers/enablers to implementing the education app from participants’ perspectives. The process evaluation will be guided by the Exploration, Preparation, Implementation and Sustainment Framework.^39^

#### App acceptability

Intervention participants will be invited to complete a user satisfaction survey after 1 month, based on previous digital health intervention trials.^29 40^ This 34-item questionnaire comprises categorical, multiple-choice and open-ended questions to explore participants’ perceptions of the app content, visuals/audio, nurse character, understandability, usefulness and functionality.

#### App feasibility

Field notes on app feasibility will be taken during recruitment. Quantitative data will also be collected to determine app feasibility, which will include (1) uptake of the intervention (% of eligible participants who enrolled in the study, noting any difficulties in enrolling and providing the app to participants on the hospital ward), (2) retention (% of enrolled participants assessed at 3-month follow-up) and (3) compliance with the intervention (% of participants who completed all six modules in the app and Google data analytics, such as time spent on each module, frequency of access to each module).

#### Barriers and enablers of implementation

Participants in the intervention group will be invited to an interview or focus group to discuss their user experience and to provide suggestions for further improving the app. These qualitative interviews are an integral component of the process evaluation and will help identify barriers and enablers to implementation. Semi-structured interviews will be conducted by a bilingual researcher, guided by a pre-established interview guide (online supplemental file 1). The interviews will be audio-recorded and analysed using directed content analysis. Maximum variation sampling will be used to obtain a range of views.

### Statistical analysis

Statistical analysis will follow the intention-to-treat principle and a prespecified statistical analysis plan led by a blinded statistician. Descriptive statistics will be used to characterise participants. The analysis of variance (ANOVA) will be used to compare the primary outcome and estimate the mean difference and 95% CI. Furthermore, to assess whether there is a change in the outcome over time (baseline, 1 month and 3 months) within and between groups, a linear mixed effects regression model will be used with a random intercept for patients to account for individual variability. Assumptions for ANOVA and the linear mixed-effects model will be assessed prior to analysis. For ANOVA, independence, normality within groups and homogeneity of variances will be checked using Levene’s test, and outliers will be addressed if necessary. For the mixed-effects model, linearity, residual normality and homoscedasticity will be evaluated through diagnostic plots. If assumptions are violated, corrective measures such as data transformation or robust methods will be applied. In the case of missing data that are missing at random in nature, multiple imputations will be used. For secondary continuous outcomes, linear mixed effects regressions will be performed to assess whether there is change in the outcomes over time within and between groups. Similarly, for secondary categorical outcomes, mixed effects logistic regression models will be used to test for the change in the outcomes over time within and between groups.

Interview audio files will be transcribed verbatim into simplified Chinese and then translated into English by a bilingual researcher. To ensure the accuracy and reliability of the translation, another bilingual author will randomly audit five translated transcripts and back-translate these from English into simplified Chinese. The data will then be analysed using directed content analysis, with coding and theme generation facilitated by an Excel spreadsheet.

### Composition, roles and responsibilities

Trial investigators will monitor enrolment, train study staff and check data quality and confidentiality. Only the investigators on the approved protocol will have access to the final dataset.

### Sample size calculation

Since there is no established minimally important difference in disease knowledge or similar intervention for the CADE-Q SV (Simplified Chinese), the sample size calculation is based on a mean change of 2.3 points (SD 3.6), which has been published in patients with CHD precompleting and postcompleting a comprehensive cardiac education programme.^41^ A total of 78 participants (39 per group) will be required to detect a between-group difference in CADE-Q SV (Simplified Chinese) score of 2.3 points with (α=0.05, 80% power) (calculated from https://sample-size.net/sample-size-means/). Accounting for a 20% dropout rate, 98 patients (49 per group) will be needed.

## Consumer and public involvement

Consumers (Chinese-speaking nurses and patients) were involved in the app adaptation process by providing individual feedback on the translated storyboard and testing the prototype. However, no consumers were involved in the design, conducting, reporting or dissemination of the study.

## Ethics and dissemination

Ethics approval for the study has been obtained from the Western Sydney Local Health District Human Research Ethics Committee (2024/STE00147). All participating hospitals have approved site-specific authorisation. All participants will be provided with detailed study information, and only those who provide written consent will be recruited (online supplemental file 2). Any protocol modifications will be communicated to the ethics committee, investigators, participants and trial registry.

The study results will be disseminated via usual scientific forums, including peer-reviewed publications, conference presentations and social media. The study participants will receive a one-page summary of the findings in lay language on request.

## Supplementary material

10.1136/bmjopen-2025-114569 online supplemental file 1

10.1136/bmjopen-2025-114569 online supplemental file 2

10.1136/bmjopen-2025-114569 online supplemental file 3
