# Supplementary material for: Effectiveness of a co-adapted virtual discharge education app on disease knowledge and health behaviours in patients following heart attack: a multicentre, randomised controlled trial protocol in Sydney, Australia
Source: BMJ Open. 2026 Feb 18;16(2):e114569. doi: 10.1136/bmjopen-2025-114569 (PMC12918686; doi:10.1136/bmjopen-2025-114569)
Supplement: online supplemental file 1 [file bmjopen-16-2-s001.docx]

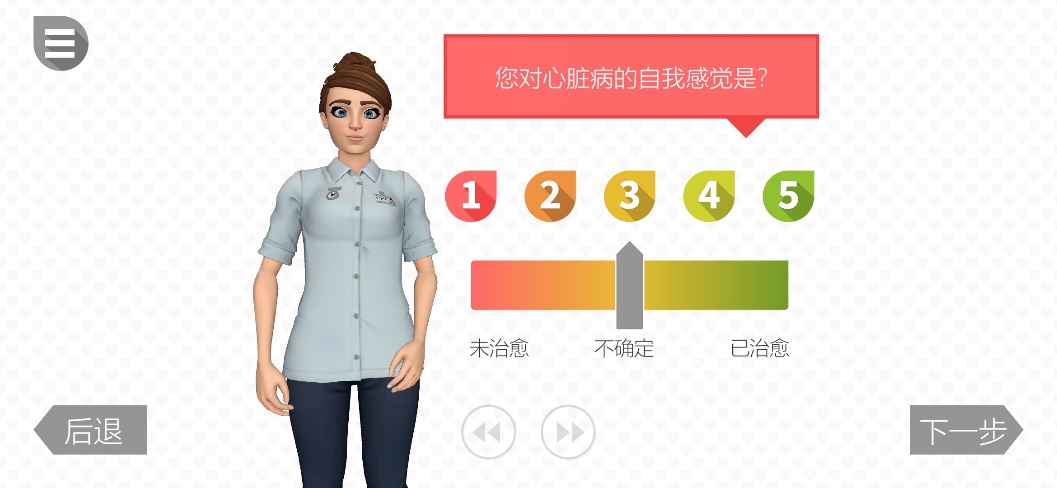


Image 1. Screenshot of the app (interacting with users via question)


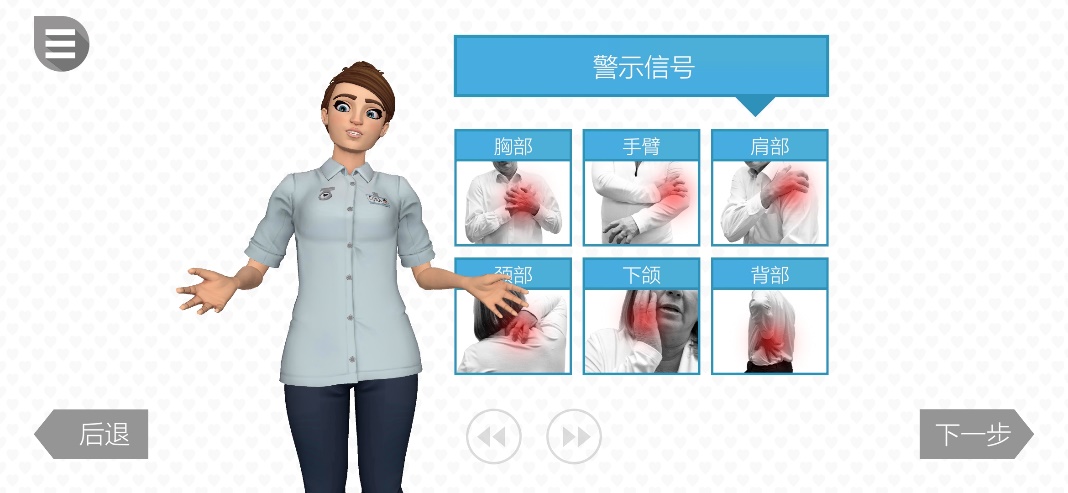


Image 2. Screenshot of the app (illustrating symptoms with images)
