# Supplementary material for: Effectiveness of a co-adapted virtual discharge education app on disease knowledge and health behaviours in patients following heart attack: a multicentre, randomised controlled trial protocol in Sydney, Australia
Source: BMJ Open. 2026 Feb 18;16(2):e114569. doi: 10.1136/bmjopen-2025-114569 (PMC12918686; doi:10.1136/bmjopen-2025-114569)
Supplement: online supplemental file 2 [file bmjopen-16-2-s002.docx]

Interview guide questions

| Thank you for taking part in this research project. We would like to learn more about your experience using this avatar-based education application and whether it has changed the way you manage your heart condition. | |
| --- | --- |
|  | Question |
| 1 | How have you got on with using the mobile app over the last three months? |
| 2 | What were the differences between the app and other patient education you received? |
| 3 | How has using the mobile app affected the way you manage your disease? |
| 4 | In what ways has the mobile app affected your everyday life? |
| 5 | What were the main benefits of using the app? |
| 6 | What were your main issues/difficulties when using the app? |
| 7 | What would you like to see changed about how you use the app? |
| 8 | How could we improve the app? |
| 9 | Do you have any other feedback? |
