# Supplementary material for: Effectiveness of a co-adapted virtual discharge education app on disease knowledge and health behaviours in patients following heart attack: a multicentre, randomised controlled trial protocol in Sydney, Australia
Source: BMJ Open. 2026 Feb 18;16(2):e114569. doi: 10.1136/bmjopen-2025-114569 (PMC12918686; doi:10.1136/bmjopen-2025-114569)
Supplement: online supplemental file 3 [file bmjopen-16-2-s003.docx]

# Participant Information and Consent Form

| Full Name of Project | A Culturally Adapted Avatar-based Discharge Education Application to Improve Disease Knowledge and Attendance at Cardiac Rehabilitation in Mandarin-speaking Patients after Acute Coronary Syndrome (ACS) |
| --- | --- |
| Short **Name of Project** | Avatar-based Discharge Education for Mandarin-speaking ACS patients |
| Protocol Number | 2024/ETH00110 |
| Project Sponsor | University of Sydney |
| Principal Investigator | Insert the name of the PI |
| Site Name | Insert the name of the site |


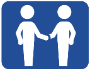


### What am I being invited to do?

The project team invites you to participate in a project that tests a new way of delivering education about your heart health and recovery. You have been invited to take part because you recently had an admission for a heart problem.

This Participant Information Sheet/Consent Form tells you about the research project. It explains the processes involved in taking part. Knowing what is involved will help you decide if you want to take part in the research.

Please read this information and feel free to ask any questions. You can take some time to make up your mind and decide if this project is right for you. You can also talk to someone you trust, like a family member, friend, or your local doctor.

If you decide you want to take part in the research project, you will be asked to sign the consent section. By signing it you are telling us that you:

• Understand what you have read

• Consent to take part in the research project

• Consent to be involved in the research described

• Consent to the use of your personal and health information as described.

You will be given a copy of this Participant Information and Consent Form to keep.


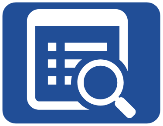


### What is the purpose of this project?

In this project, we will investigate the effectiveness of an avatar-based education application (app). After a heart attack or event, it is important to take any new medications and make changes to your lifestyle to prevent another heart attack, even if you have had treatment such as surgery or a stent. This app will take you through 6 education modules based on information from the Heart Foundation. This app is delivered in Mandarin and involves videos, games, and quizzes to help you learn how to make healthy changes for your heart.

This education application has been effective in improving knowledge in a small group of English-speaking people after a heart attack. We aim to test the effectiveness of the Mandarin version of the app in a larger Mandarin-speaking group. Therefore, a total of 78 participants will be recruited from three hospitals. If successful, we can offer this app to all Mandarin-speaking people after a heart attack before they leave the hospital.

This research project is a collaboration between Hospitals in Local Health Districts, NSW and the University of Sydney.


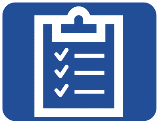


### What do I have to do if I take part?

One of the project team members will provide information about the project to you to see if you are interested in being involved. They will answer any questions you have about the project.

If you agree to take part in this project, we will ask you to read and sign a consent form before any study activity occurs. After signing the consent form you will be given a unique participant study ID so that your name and other information are not used. This ID will be used for all project paperwork to protect your privacy.

This is a 3-month randomised controlled research project. Sometimes we do not know which treatment is best for patients, so we need to compare the education app with usual hospital care to find out. We put people into two groups and give each group a different treatment. The results are compared to see if one is better. To try to make sure the groups are the same, each participant is put into a group by chance (random). You have a one-in-two chance of being in the education app group.

If you are randomized to the education app group, a researcher will assist you in downloading the app to your personal smartphone or tablet device. If you have difficulty downloading the app while in the hospital, you will be contacted by phone or email after discharge to ensure the app is set up correctly. You will have unlimited access to the app as many times as you like for the duration of the study. You will independently work through the six modules containing videos, animations, games, and education from the avatar nurse. Each module concludes with a quiz. Approximately 40 minutes is required to complete all the modules.

Regardless of which group you are randomly allocated to, you will be asked to complete some questionnaires about your knowledge of heart disease and medications, your smoking status, physical activity habits and self-care behaviours. We will ask you to complete questionnaires before discharge from the hospital and follow-up at 1 month and 3 months after discharge. There are 7 questionnaires we would like you to complete at different times during the project.

This table below outlines what you need to do in this project. For more information, please ask a member of the project team.

| **What part of the project?** | **What do I have to do?** |
| --- | --- |
| Consenting to take part in this project | If you are happy to take part in this project, you will be asked to sign a consent form. |
| When you start the project | Before the hospital discharge, you will be asked to complete some questionnaires in person, which takes about 20 minutes. |
| During the project | You will be asked to complete some questionnaires via phone, online or face-to-face as you prefer 1 month after discharge, which takes about 20 minutes. |
| At the end of the project | You will be asked to complete some questionnaires via phone, online or face-to-face as you prefer 3 months after discharge, which takes about 20 minutes. |

You can stick this on your refrigerator to see when your follow-up calls are and what is involved at each follow-up.

There may be further work on this project, such as participation in an interview or focus group. If you are agreeable to this, please sign the specific section of the consent form pertaining to this.

There are no additional costs associated with participating in this research project, nor will you be paid. All-access to the education app as part of the research project will be provided to you free of charge.


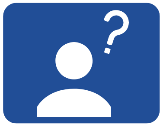


### Do I have to take part and can I change my mind?

**Taking part is up to you**

Participation in any research project is completely voluntary. You get to decide whether you take part in this project. You can say yes or no.

Your decision won’t affect your routine treatment and your relationship with those treating you, such as doctors, nurses, and other healthcare providers, or your relationship with the hospital or researchers.

**You can change your mind at any time**

If you do take part, you can stop at any time. If you want to stop, please tell someone in the project team. You do not have to tell us the reason.

Once you stop taking part, we will not collect any more information about you. We will keep the information we have already collected to make sure the results of the project can be measured properly.

Your decision to stop taking part won’t affect your routine treatment, relationship with those treating you, such as doctors, nurses, and other healthcare providers, or your relationship with the hospital or researchers.

**The project might stop for other reasons**

We do not anticipate stopping the study unexpectedly. We might need to stop the project while you are taking part for unforeseen circumstances. If this happens, we will explain the reasons to you.

We may also ask you to stop taking part in the project if it is no longer in your best interest. If this happens, we will discuss this with you.


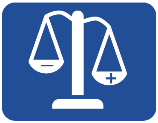


### What are the possible benefits of taking part?

You may not directly benefit from taking part in this project. However, we imagine that using the education app we are testing in this study may improve your knowledge of heart disease and behaviours linked to your heart health.

By taking part, you will help the researchers understand more about the role of the avatar-based app in heart disease patient education. This knowledge may help people in the future.


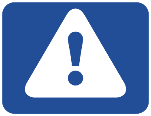


### What are the possible risks and disadvantages of taking part?

There are no physical, social, or economic risks or harms anticipated from participating in this project.

However, your participation in this project requires completing initial and follow-up questionnaires in addition to usual care. Each assessment is anticipated to take less than 30 minutes. You may feel that some questions in the questionnaire/survey/interview are sensitive, and this may cause you distress. If this happens, you can take a break from/stop the questionnaire/survey/interview at any time. We can provide someone qualified and not part of this project team to support you if you wish. This counselling will be provided free of charge.

**Breach of confidentiality**

In focus groups, we will talk about topics related to you. There is a chance that other people in the group could share information from the focus group with others outside of this project. We will remind everyone who takes part that they must keep what they hear in this focus group confidential and not share it with others.


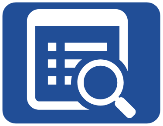


### What happens when the research project ends?

Your involvement in the study ends after completing the 3-month follow-up assessment. We anticipate that the results of the study will be available six months after the final assessment. You may contact the Research Contact Person on page 7 of this form to be sent a copy of the study findings if you wish. If you received the app at the beginning of the study, you will continue to have access to it for no cost. If you expressed interest in receiving the app during your baseline assessment but were not allocated to the intervention group, a link to the app installation will be sent to you upon completion of your final assessment. Once you install the app, you will have free access to the app. Along with the link, a survey about your satisfaction with the app’s content and usability will be sent to you, which we encourage you to complete after using the app.


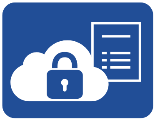


### If I take part, what will happen to my information?

**Collecting your information**

By signing the consent form, you consent to the relevant research team collecting and using personal information about you for the research project. We will collect information for this project from medical records and you. Any information obtained in connection with this research project that can identify you will remain confidential. To protect your privacy, the information will be linked to a unique code, also known as an identification number, that is created for you. We will not link your identification number to your name or contact information. Your information will only be disclosed with your permission, except as required by law. Only approved project team members will have access to your personal contact details.

**Keeping your information safe**

To keep your information safe, we will:

follow all relevant privacy requirements

keep it secure. Your consent form, with your name on it, and all study paper documents will be scanned at each participating site (Westmead, St George and RPA Hospitals) and uploaded into REDCap. Once the paper records are transferred to an electronic version, they will be securely shredded at each participating site. All electronic data will be stored on REDCap under the University of Sydney licence - a secure password-protected web platform. The scanned consent forms will be stored in a database separated from the database where data will be held. No one can identify you from your data except for research team members who have special approved access.

take steps to prevent anyone from accessing information that identifies you unless they need to, for example, check it in an audit.

give it a code and keep it separate from anything that could easily identify you, like your name or contact information.

You can ask us to tell you what information we have collected about you as part of this project. If your information is not correct, you can also ask us to change it. Please contact the study team member named at the end of this document if you would like to access your information.

We will keep your information for 5 years after the results of the project are published. After this, we will delete data from the electric database.

It is anticipated that the results of this research project will be published and/or presented in a variety of forums. In any publication and/or presentation, information will be provided in such a way that you cannot be identified, except with your permission. All data will be presented using participant codes.

**Sharing your information with others**

We may share some of your information with others.

**Sharing information with your clinicians**: If we find information relevant to your ongoing care, such as the identification of a new risk factor, we will share this information with your clinicians, such as cardiologists and nurses, so you can receive the care you need.

**Sharing information with other researchers:** we may share certain information from this project so that other researchers can use it in the future. These researchers may be in Australia or overseas. We will only share information that has been aggregated (that is, joined together with information from others before sharing) to ensure anonymisation.


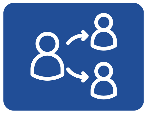


### How may my information be shared in the future?

We will ask you to consider sharing your information for future research. Sharing information with others can help make all research more effective.

When we share your information, we will take steps to make it difficult for anyone to link the information back to you. This includes removing information that could easily identify you, like your name or contact information. There is still a very small chance that someone could identify you again.

You can choose the kinds of research for which we share your information:

Any future research

Research projects that are closely related to this one

You will not be told about future research projects if you agree to share your information. If you change your mind, you have the option to ask us to stop sharing your information. However, if your information has already been shared, it may not be possible to retrieve or destroy it.


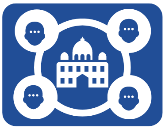


### Who is running and paying for this project?

This research is a collaboration between (insert the name of the hospital), the University of Sydney and Flinders University. Dr Ling Zhang is funded by a SOLVE-CHD NHMRC Synergy Grant for her post-doctoral position.

This project is being funded by the Bright Ideas Grant provided by the Faculty of Medicine and Health, University of Sydney. Access to the avatar app is provided by Flinders University.

If knowledge acquired through this research leads to discoveries that are of commercial value to Westmead Hospital, the University of Sydney or the study researchers, there will be no financial benefit to you or your family from these discoveries.

No research team member will receive a personal financial benefit from your involvement in this research project (other than their ordinary wages).


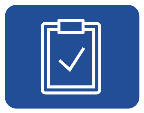


### Who has approved this project?

The HREC of Western Sydney Local Health District (WSLHD) approved this project. This committee ensures that this project meets Australian ethical standards for research involving people.

This project will be carried out according to the National Statement on Ethical Conduct in Human Research (2007). This statement has been developed to protect the interests of people who agree to participate in human research studies.


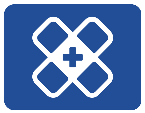


### What happens if something goes wrong?

If you are harmed because of taking part in this project, in an emergency, you should call 000 or go to the emergency department at your nearest hospital. If you suffer any distress or psychological injury as a result of this research project, you should contact the study team as soon as possible. You will be assisted with arranging appropriate treatment and support.


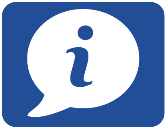


### Where can I find more information and who to contact?

Thank you for taking the time to read this information about our project. The person you may need to contact will depend on the nature of your query.

**Research contact person**

You can contact a project team member, or the research members listed below at any time to ask questions about the project and your involvement in the project.

| Name | Role | Phone number | Email |
| --- | --- | --- | --- |
| Ling Zhang | Project Coordinator | 0434209383 | ling.zhang1@sydney.edu.au |
| Wendy Shi | Research Team Member | 0450714809 | wshi9292@uni.sydney.edu.au |

**Complaints contact person**

For matters relating to research at the site at which you are participating, the details of the local site complaints person are listed below. (Insert the details of the relevant local site)

| Position |  |
| --- | --- |
| Telephone |  |
| Email |  |

If you have any complaints about the ethics of the project, the way it is being conducted or any questions about being a research participant in general, then you may contact:

**Reviewing HREC approving this research and HREC Executive Officer details**

| Reviewing HREC name | WSLHD Human Research Ethics Committee |
| --- | --- |
| HREC Executive Officer | Kellie Hansen |
| Telephone | 02 8890 9007 |
| Email | wslhd-researchoffice@health.nsw.gov.au |

**Research Governance Officer details** (Insert the details of the relevant local site)

| Position | Research Governance Officer |
| --- | --- |
| Telephone |  |
| Email |  |

# Signature Page

| Full Name of Project | A Culturally Adapted Avatar-based Discharge Education Application to Improve Disease Knowledge and Attendance at Cardiac Rehabilitation in Mandarin-speaking Patients after Acute Coronary Syndrome (ACS) |
| --- | --- |
| Short **Name of Project** | Avatar-based Discharge Education for Mandarin-speaking ACS patients |
| Protocol Number | 2024/ETH00110 |
| Project Sponsor | University of Sydney |
| Principal Investigator | Insert the name of the PI |
| Site Name | Insert the name of the site |

**Person taking part in the project**

Signature: ______________________________________________ Date: ______________

Name: _________________________________________________

**Person conducting the informed consent discussion**

I have explained the research project, its procedures and risks to the participant and I believe they have understood that explanation.

Signature: ______________________________________________ Date: ______________

Name: __________________________________

Each person must sign and personally date this consent form

# Form for Withdrawal of Participation

| Full Name of Project | A Culturally Adapted Avatar-based Discharge Education Application to Improve Disease Knowledge and Attendance at Cardiac Rehabilitation in Mandarin-speaking Patients after Acute Coronary Syndrome (ACS) |
| --- | --- |
| Short **Name of Project** | Avatar-based Discharge Education for Mandarin-speaking ACS patients |
| Protocol Number | 2024/ETH00110 |
| Project Sponsor | University of Sydney |
| Principal Investigator | Insert the name of the PI |
| Site Name | Insert the name of the site |

**Declaration by Participant**

I wish to withdraw from participation in the above research project and understand that such withdrawal will not affect my routine treatment, my relationship with those treating me or my relationship with Westmead Hospital.

|  | | | | | | |
| --- | --- | --- | --- | --- | --- | --- |
|  | Name of Participant (please print) | |  |  |  |  |
|  | | | | | | |
|  | Signature |  | | Date |  |  |
|  | | | | | | |

In the event that the participant’s decision to withdraw is communicated verbally, the Senior Researcher will need to provide a description of the circumstances below.

|  |
| --- |

**Declaration by Researcher^†^**

I have given a verbal explanation of the implications of withdrawal from the research project and I believe that the participant has understood that explanation.

|  | | | | | | |
| --- | --- | --- | --- | --- | --- | --- |
|  | Name of Researcher (please print) | |  | | |  |
|  | | | | | |  |
|  | Signature |  | | Date |  |  |
|  | | | | | | |

^†^ An appropriately qualified member of the research team must provide the explanation of, and information concerning, the research project.

Note: All parties signing the consent section must date their own signature.
