# Supplementary material for: Effectiveness of a co-adapted virtual discharge education app on disease knowledge and health behaviours in patients following heart attack: a multicentre, randomised controlled trial protocol in Sydney, Australia
Source: BMJ Open. 2026 Feb 18;16(2):e114569. doi: 10.1136/bmjopen-2025-114569 (PMC12918686; doi:10.1136/bmjopen-2025-114569)
Supplement: Supplementary file 4 [file bmjopen-2025-114569.reviewer_comments.pdf]

PEER REVIEW HISTORY

BMJ Open publishes all reviews undertaken for accepted manuscripts. Reviewers are asked to complete a checklist review form and are provided with free text boxes to elaborate on their assessment. These free text comments are reproduced below.

ARTICLE DETAILS

Title (Provisional)

The effectiveness of a co-adapted virtual discharge education app on disease knowledge and health behaviours in patients following a heart attack: a multicentre, randomised controlled trial protocol in Sydney, Australia

Authors

Zhang, Ling; Shi, Wendan; Zhao, Emma; Hyun, Karice K; Zecchin, Robert; Gao, Yan; Brunorio, Ludimila; Stanaway, Fiona; Ellis, Tiffany; Redfern, Julie; Clark, Robyn; Du, Huiyun; Gallagher, Robyn

---

This article was not externally reviewed at BMJ Open. Protocols that have been independently assessed prior to submission to BMJ Open are usually fast-tracked to publication on the grounds that further substantial changes will not be possible. This independent assessment will usually be external, independent review for both a substantial grant award from a non-commercial or government funder and ethics approval.
